# Prevalence of Dental Caries and Periodontal Disease, Access to Dental Services and Perception of Oral Health in Adolescents and Adults from a Rural Community in Angola

**DOI:** 10.3290/j.ohpd.b5877397

**Published:** 2024-12-12

**Authors:** Marcial António Simão Songa, Tânia Adas Saliba, Nemre Adas Saliba, Fernando Yamamoto Chiba, Suzely Adas Saliba Moimaz

**Affiliations:** a Marcial António Simão Songa PhD Student, São Paulo State University (UNESP), School of Dentistry, Araçatuba, Brazil. Data collect, wrote the manuscript, reviewed the final version of the manuscript, substantial contribution to the discussion.; b Tânia Adas Saliba Associate Professor, São Paulo State University (UNESP), School of Dentistry, Araçatuba, Brazil. Data analysis, wrote the manuscript, reviewed the final version of the manuscript, substantial contribution to the discussion.; c Nemre Adas Saliba Full Professor, São Paulo State University (UNESP), School of Dentistry, Araçatuba, Brazil. Study idea, experimental design, data analysis, wrote the manuscript, reviewed the final version of the manuscript, substantial contribution to the discussion.; d Fernando Yamamoto Chiba Assistant Professor, São Paulo State University (UNESP), School of Dentistry, Araçatuba, Brazil. Data analysis, manuscript writing, review of the final version of the manuscript, substantial contribution to the discussion.; e Suzely Adas Saliba Moimaz Full Professor, São Paulo State University (UNESP), School of Dentistry, Araçatuba, Brazil. Study idea, experimental design, data collection, data analysis, wrote the manuscript, reviewed the final version of the manuscript, substantial contribution to the discussion.

**Keywords:** adolescent, adult, Angola, dental caries, health services accessibility

## Abstract

Purpose: To investigate the epidemiological profile of dental caries and periodontal disease, access to dental services, and perception of oral health in adolescents and adults from a rural community in Angola.

Materials and Methods: This is an observational, analytical and cross-sectional study, performed with 575 individuals aged between 12 and 40 years. The prevalence of caries and periodontal disease was assessed using the DMFT index and the Community Periodontal Index. Data on access to dental services, health and oral hygiene habits, and perception of oral health were collected through interviews.

Results: 42.8% never had a dental appointment; 85.1% had their last consultation in a public health service; 60.2% considered the service to be average/poor; 32.5% had their last consultation due to pain; 57.4% considered their oral health to be good/very good; 51.0% brushed their teeth twice a day; and 36.9% did not use toothpaste. The prevalence of untreated caries was 72.9% and only 1.7% of teeth affected by tooth decay were restored. A mean of 0.88 ± 1.44 sextants showed gingival bleeding; 1.46 ± 1.74 showed dental calculus; and 0.16 ± 0.58 showed periodontal pockets. The prevalence of sextants with periodontal pockets of 6 mm or more was 1.7%.

Conclusion: The prevalence of untreated caries was high, while periodontal disease does not represent a severe problem in this population. Access to dental services is poor and limited to extractions.

According to the World Health Organization, oral diseases affect approximately 3.5 billion people worldwide, making it one of the most prevalent non-communicable diseases, especially in low- and middle-income countries.^22^ In this context, marked inequalities in the distribution of the burden of oral diseases and access to oral health care, especially among disadvantaged and marginalised population groups, represents major challenges to health systems.^22,23^

The African Region of the World Health Organization consists of 47 countries and has a population of around 1.1 billion inhabitants. In the period between 1990 and 2019, the African Region recorded the largest increase in the number of serious cases of oral diseases compared to other regions of the world. Given this scenario, the African Regional Committee of the World Health Organization approved, during its 66th session, the Regional Oral Health Strategy 2016–2025, with the aim of reducing non-communicable diseases and related risk factors, and promoting actions to prevent and control oral diseases for all people in the African Region.^24^

The fragility of the oral health condition of the African population has been highlighted in the literature.^2,5^ An epidemiological study carried out with school-age children living in the peri-urban area of the city of Benguela, Angola, found a critical situation regarding the prevalence of caries in the primary dentition, highlighting the limited access to dental services and the high number of untreated carious lesions.^17^ A precarious oral health situation was also identified in research carried out with elderly residents of the municipality of Bocoio, Angola, in which a high number of lost teeth and deficiencies in prosthetic rehabilitation were found.^4^

As an aggravating factor in Africa, in addition to the unequally distributed burden of oral diseases, there is also a great lack of epidemiological data on the oral health of population groups in situations of greater vulnerability.^1^

Given the above, the objective of this study was to investigate the epidemiological profile of caries and periodontal disease, access to dental services and the perception of oral health in adolescents and adults in a rural community in Angola.

## MATERIALS AND METHODS

The recommendations of the STROBE protocol were followed for the preparation of this manuscript.^19^ This is an observational, analytical and cross-sectional epidemiological study, carried out with adolescents and adults in the city of Benguela, Angola, in 2023.

The study included individuals aged 12 to 40 years, of both sexes, living in a rural location in the province of Benguela, who signed the Free and Informed Consent Form. In the case of individuals under the age of 18, the Free and Informed Consent Form was obtained from their legal guardians and the Free and Informed Assent Form from the participant. Individuals who had physical limitations that made it impossible to perform the oral clinical examination and those who were absent after five data collection attempts were excluded from the study.

The study population consisted of all individuals aged 12 to 40 years, residing in the Asseque region, a rural location in the province of Benguela. The list of the estimated number of inhabitants in the region was obtained through meetings with the head of the region’s Health Post, the local community leader, and the zone administrator, totaling 4655 inhabitants. Based on this information, a list of all existing residences in the Asseque region was obtained. To recruit participants, all residences located in the region were visited and all residents, in accordance with the inclusion and exclusion criteria adopted, were invited to participate in the study.

The Asseque region was selected to carry out the study, considering the characteristics of the population living in rural areas, the distance from the city center and the lack of dental services at the locality. The sample size calculation was carried out based on data from a pilot study conducted on individuals not included in the final study sample, considering the prevalence of caries as the primary outcome. The minimum sample size was calculated according to the estimated total number of individuals aged 12 to 40 living in the province of Benguela, adopting an acceptable margin of error of 5% and a confidence level of 95%. Therefore, the minimum sample size determined was 357 individuals. All individuals aged 12 to 40 years living in this region were invited to participate in the study. The overall response rate of the clinical examination and questionnaire was 92%. Of the total of 625 individuals identified who met the sample inclusion and exclusion criteria, 575 agreed to participate in the study. No participant was edentulous.

Information on sociodemographic characteristics, use of dental services, health and oral hygiene habits, and perception of oral health were collected through interviews using a semi-structured questionnaire.

To assess the prevalence and severity of caries and periodontal disease, the DMFT index and the Community Periodontal Index were used, respectively. In this study, as diagnostic criteria, caries was recorded as present when a lesion in a groove, fissure, or on a smooth surface of the tooth had an evident cavity, unsupported enamel, or a detectably softened bottom or wall. A tooth with a temporary restoration or that bore a dental sealant but was also decayed, was considered decayed. The WHO millimeter periodontal probe was used to confirm visual evidence of tooth decay. The periodontal condition was assessed using a WHO millimeter periodontal probe. The mouth was divided in sextants and the teeth 17, 16, 11, 26, 27, 37, 36, 31, 46 and 47 were examined and subsequently evaluated for the occurrence of gingival bleeding, presence of supra- and subgingival calculus, and periodontal pockets with probing depths between 3.5–6.0 mm.^25^ The presence of alterations in oral tissues was also investigated.

Data were collected by a single team composed of an examiner and a note-taker, previously trained and calibrated. When calculating the simple Kappa coefficient, a degree of intra-examiner agreement of 0.92 was obtained for the analysis of caries and also for the analysis of periodontal diseases. The researchers’ calibration process was carried out following all stages of theoretical discussion of the variables used, codes and examination criteria; practical discussion; carrying out examinations to perform the calibration; final discussion; and calculation of the simple Kappa coefficient. Regarding the analysis of dental condition to assess caries, the unit of analysis adopted was the dental element, with verification of the conditions: healthy tooth; decayed tooth; tooth filled and with cavities; tooth filled and free of cavities; and tooth missing due to caries. Regarding the analysis of periodontal diseases, the unit of analysis adopted was the oral sextant, with verification of the conditions: healthy sextant; sextant with gingival bleeding; sextant with dental calculus; sextant with periodontal pocket of 4 to 5 mm; and sextant with a periodontal pocket of 6 mm or more.

The exams were carried out in the participants’ homes, in an adequately ventilated place with natural light, using a WHO millimeter periodontal probe and a flat mouth mirror. Sterile gauze was used to help clean and dry the teeth. The examiner adopted a systematic approach to assessing the condition of the tooth. The examination was carried out in an orderly manner, proceeding from one tooth or tooth space to the adjacent tooth or tooth space. A tooth was considered present in the mouth when any part of it was visible. If a permanent and a primary tooth occupied the same tooth space, only the condition of the permanent tooth was recorded. Third molars were included in the examination.

The data were analysed using descriptive statistics techniques and the results expressed through tables. The associations between the characteristics of the last dental appointment, self-perception of oral health, health habits, oral clinical aspects, oral hygiene characteristics and age group were analysed using the chi-squared test and the G-Test.

Data normality was analysed using the D’Agostino-Pearson test. Between-group comparisons of the DMFT index, number of decayed teeth, number of missing teeth, number of restored teeth, number of healthy sextants, number of sextants with gingival bleeding, number of sextants with dental calculus and number of sextants with periodontal pockets were performed using the Kruskal-Wallis test. Comparison between genders was performed using the Mann-Whitney test. Data processing and analysis were carried out using EpiInfo software version 7.2.2, adopting a significance level of 5%.

The study was carried out in accordance with the ethical principles of the Declaration of Helsinki and was approved by the Ethics and Research Committee of the Higher Polytechnic Institute of Benguela (Process number: 01/DCTS/ISPB/2023).

## RESULTS

Of the total of 575 participants, 56.2% were male and 43.8% were female, with an average age of 22.36 ± 9.07 years.

As seen in Table 1, 42.8% of participants had never had a consultation with a dentist, especially individuals aged 12–18 years (p < 0.0001). Among the participants who had attended the dentist, the majority reported that their last consultation was carried out at a public health/dental service (85.1%) and considered the quality of care as acceptable, bad or very bad (60.2%). Approximately one-third of participants (32.5%) had their last dental appointment due to pain. The majority of individuals considered their oral health to be good or very good (57.4%); however, a statistically significant proportion of individuals aged 30–40 years (21.4%) considered their oral health to be poor (p < 0.0001). The data presented in Table 1 demonstrate that the habits of smoking and drinking alcohol frequently/daily was reported by 5.9% and 5.7% of participants, respectively. A statistically significant association (p < 0.0001) was noted between not smoking and being 12–18 years of age, while there was a statistically significant association (p < 0.0001) between consuming alcoholic beverages frequently/daily and being 30–40 years of age. The majority of participants did not have halitosis (79.0%) or changes in the oral mucosa (91.1%), brushed their teeth twice a day (51.0%), used a conventional toothbrush (74.4%) and toothpaste (63.1%).

**Table 1:** Characteristics of the last dental appointment, self-perception of oral health, health habits, oral clinical aspects and oral hygiene characteristics in adolescents and adults, according to age group in Benguela, Angola, 2023

Variable	Age range	p-value
12–18 years	19-29 years	30-40 years	Total
Gender	n	%	n	%	n	%	n	%	0.1336#
Male	150	60.98	102	52.58	71	52.59	323	56.17
Female	96	39.02	92	47.42	64	47.41	252	43.83
Total	246	100.00	194	100.00	135	100.00	575	100.00
Time since last dental appointment	n	%	n	%	n	%	n	%	< 0.0001#
Less than 1 year	50	20.33	73	37.63	50	37.04	173	30.09
Between 1 and 2 years	19	7.72	23	11.86	13	9.63	55	9.57
3 years or more	22	8.94	40	20.62	39	28.89	101	17.57
Never been to the dentist	155	63.01	58	29.9	33	24.44	246	42.78
Total	246	100.00	194	100.00	135	100.00	575	100.00
Location of last dental appointment	n	%	n	%	n	%	n	%	0.5212*
Public service	80	87.91	118	86.76	82	80.39	280	85.11
Private service	6	6.59	8	5.88	13	12.75	27	8.21
Faculty	2	2.2	3	2.21	4	3.92	9	2.74
Others	3	3.3	7	5.15	3	2.94	13	3.95
Total	91	100.00	136	100.00	102	100.00	329	100.00
Reason for last dental appointment	n	%	n	%	n	%	n	%	0.1156#
Prevention consultation	54	59.34	88	64.71	51	50	193	58.66
Pain	26	28.57	39	28.68	42	41.18	107	32.52
General treatment	11	12.09	9	6.62	9	8.82	29	8.81
Total	91	100.00	136	100.00	102	100.00	329	100.00
Perception about the quality of care at the last dental appointment	n	%	n	%	n	%	n	%	0.1879#
Very good/good	34	37.36	47	34.56	50	49.02	131	39.82
Regular	21	23.08	34	25	16	15.69	71	21.58
Very bad/bad	36	39.56	55	40.44	36	35.29	127	38.6
Total	91	100.00	136	100.00	102	100.00	329	100.00
Self-perception of oral health	n	%	n	%	n	%	n	%	< 0.0001#
Very good/good	140	56.91	123	63.4	67	49.63	330	57.39
Regular	94	38.21	54	27.84	39	28.89	187	32.52
Bad	12	4.88	17	8.76	29	21.48	58	10.09
Total	246	100.00	194	100.00	135	100.00	575	100.00
Smoking habit	n	%	n	%	n	%	n	%	< 0.0001#
No	246	100.00	174	89.69	121	89.63	541	94.09
Yes	0	0	20	10.31	14	10.37	34	5.91
Total	246	100.00	194	100.00	135	100.00	575	100.00
Alcohol consumption	n	%	n	%	n	%	n	%	< 0.0001#
Never/rarely	245	99.59	180	92.78	117	86.67	542	94.26
Several times a month/frequently	1	0.41	11	5.67	16	11.85	28	4.87
Several times a week/daily	0	0	3	1.55	2	1.48	5	0.87
Total	246	100.00	194	100.00	135	100.00	575	100.00
Halitosis	n	%	n	%	n	%	n	%
Never/rarely	203	82.52	153	78.87	98	72.59	454	78.96	0.1871*
Sometimes/often	41	16.67	39	20.1	33	24.44	113	19.65
Every day	2	0.81	2	1.03	4	2.96	8	1.39
Total	246	100.00	194	100.00	135	100.00	575	100.00
Alterations in the oral mucosa	n	%	n	%	n	%	n	%	0.0577#
No	231	93.9	176	90.72	117	86.67	524	91.13
Yes	15	6.1	18	9.28	18	13.33	51	8.87
Total	246	100.00	194	100.00	135	100.00	575	100.00
Frequency of toothbrushing	n	%	n	%	n	%	n	%	0.3026*
Do not brush/brush rarely	3	1.22	5	2.58	1	0.74	9	1.57
Once a day	82	33.33	72	37.11	40	29.63	194	33.74
Twice a day	127	51.63	87	44.85	79	58.52	293	50.96
Three times a day	34	13.82	30	15.46	15	11.11	79	13.74
Total	246	100.00	194	100.00	135	100.00	575	100.00
Item used to perform toothbrushing	n	%	n	%	n	%	n	%	0.3274*
Traditional toothbrush	185	75.2	145	74.74	98	72.59	428	74.43
Ecological toothbrush	59	23.98	45	23.2	30	22.22	134	23.3
Wooden or plastic toothpick	2	0.81	3	1.55	6	4.44	11	1.91
Finger	0	0	1	0.52	1	0.74	2	0.35
Total	246	100.00	194	100.00	135	100.00	575	100.00
Use of toothpaste	n	%	n	%	n	%	n	%	0.0877#
No	79	32.11	82	42.27	51	37.78	212	36.87
Yes	167	67.89	112	57.73	84	62.22	363	63.13
Total	246	100.00	194	100.00	135	100.00	575	100.00
#Chi-squared test; *G-test

The DMFT index of the analysed participants was 4.64 ± 5.51. It was found that only 26.3% (n = 151) of participants were caries-free, corresponding to 42.7% (n = 105) of individuals aged 12–18 years, 18.0% (n = 35) of individuals between 19 and 29 years of age, and 8.2% (n = 11) of individuals aged 30–40 years. The overall prevalence of untreated caries was 72.9% (Table 2). The prevalence of untreated caries was 53.2% among 12- to 15-year-olds; 68.6% among 16- to 19-year-olds; 83.3% in the 20–29 age group; and 88.9% among those aged 30–40 years.

**Table 2:** DMFT index and its componentes, oral sextants according to periodontal condition and prevalence of untreated caries in adolescents and adults in Benguela, Angola, 2023

Variable	Mean ± SD
DMFT Index	4.64+5.51
Decayed teeth	3.91+4.49
Missing teeth	0.65+2.70
Filled teeth	0.08+0.82
Healthy sextants	4.10+2.06
Sextants with gum bleeding	0.88+1.44
Sextants with dental calculus	1.46+1.74
Sextants with periodontal pocket	0.16+0.58
Variable	Prevalence (%)
Individuals with untreated caries	72.87


As shown in Table 3, a statistically significant difference was found in the DMFT index (p < 0.05) between all age groups analysed, with an increase in the index value as the age group increased. The number of decayed teeth was statistically significantly lower (p < 0.05) among the of 12- to 18-year-olds compared to individuals in the age groups of 19–29 years and 30-40 years. The number of missing teeth was statistically significantly higher (p < 0.05) in the 30- to 40-year-old age group compared to the 12- to 18-year-olds and 19- to 29-year-old age group. There was no statistically significant difference in the number of teeth filled between age groups. In all age groups, it was observed that a reduced proportion of teeth affected by caries were restored, representing 1.7% of the total teeth affected.

**Table 3:** Average DMFT index and its components in adolescents and adults, according to age group in Benguela, Angola, 2023

Variable	Age range
12–18years	19–29years	30–40years
DMFT Index	2.35+3.45^a^	4.87+4.69^b^	8.47+7.23^c^
Decayed teeth	2.32+3.43^a^	4.42+4.00^b^	6.07+5.65^b^
Missing teeth	0.03+0.25^a^	0.44+2.31^a^	2.10+4.53^b^
Filled teeth	0.01+0.13^a^	0.01+0.07^a^	0.31+1.66^a^
Different letters indicate a statistically significant difference (p < 0.05) between age groups.

Among the total number of participants examined, there was an average of 0.88 ± 1.44 sextants with gingival bleeding, 1.46 ± 1.74 sextants with dental calculus and 0.16 ± 0.58 sextants with periodontal pockets per individual. The overall prevalence of individuals with periodontal pockets of 6 mm or more was 1.7% (Table 4).

**Table 4:** Average number of oral sextants examined in adolescents and adults, according to periodontal condition and age group in Benguela, Angola, 2023

Periodontal Condition	Age range
12–18years	19–29years	30–40years
Healthy sextants	4.15+2.02^a^	4.29+1.97^a^	3.76+2.21^a^
Sextants with gum bleeding	0.73+1.21^a^	0.81+1.38^a^	1.17+1.73^a^
Sextants with dental calculus	1.45+1.69^a^	1.37+1.72^a^	1.60+1.84^a^
Sextants with periodontal pocket	0.03+0.16^a^	0.11+0.42^ab^	0.40+0.94^b^
Variable	Prevalence (%)
Individuals with sextants with periodontal pocket of 6 mm or more	1.74
Different letters indicate a statistically significant difference (p < 0.05) between age groups.

Table 4 shows that there was no statistically significant difference in the number of healthy sextants, sextants with gingival bleeding and sextants with dental calculus between age groups. The number of sextants with periodontal pockets was statistically significantly lower (p < 0.05) in the age group of 12–19 years compared to individuals in the age groups of 12–18 and 30-40 years. The prevalence of periodontal pockets of 6 mm or more was 0.9% among the 12- to 15-year-olds, 0% among 16- to 19-year-olds, 1.2% in the age group 20-29, and 5.5% in the 30–40 age group.

By gender, there were no statistically significant differences in the DMFT index (men: 4.31 ± 5.14; women: 5.05 ± 5.95; p =  0.1546); number of decayed teeth (men: 3.67 ± 4.19; women: 4.20 ± 4.83; p = 0.2115), missing teeth (men: 0.58 ± 2.44; women: 0.74 ± 2.99; p = 0.71), and filled teeth (men: 0.06 ± 0.72; women: 0.11 ± 0.92; p = 0.6449). Regarding periodontal condition, the comparison by gender did not demonstrate statistically significant differences in the number of sextants with gingival bleeding (men: 0.92 ± 1.48; women: 0.82 ± 1.39; p = 0.66), sextants with periodontal pockets of 4 to 5 mm (men: 0.08 ± 0.36; women: 0.18 ± 0.62; p = 0.3489), and sextants with periodontal pockets of 6 mm or more (men: 0.05 ± 0.39; women: 0.02 ± 0.14; p = 0.9494). The number of sextants with dental calculus was statistically significantly higher (p = 0.0228) in men (1.63 ± 180) compared to women (1.23 ± 1.64).

## DISCUSSION

The findings of the present study demonstrated that the population investigated has a severe deficiency in access to dental services, characterised by a large proportion of individuals who have never had a consultation with a dentist, especially among adolescents. Additionally, it was possible to verify that, when it existed, access to dental services occurred mainly through the public health/dental service and was limited to mutilating extraction treatments.

The scarcity of trained human resources is a factor of great relevance in the context of access to dental services. A study carried out with all 47 member states of the African Region of the World Health Organization analysed the oral health workforce, identifying an unequal distribution of the same mainly between urban and rural areas, as well as the low priority given to oral healthcare as the main challenges to be faced. This highlights the importance of strategies and actions to strengthen oral health policies and encourage work in areas with greater deficiency in the workforce.^6^

The inadequate proportion of dental surgeons in relation to the number of inhabitants of Angola and the scarcity of undergraduate and postgraduate courses in dentistry are also factors that may be related to the difficulty in accessing dental services. In this context, a study demonstrated that, in 2019, Angola had only 701 dentists regularly registered to serve a population of approximately 30,175,553 inhabitants, equivalent to the proportion of 1 professional for every 43,460 inhabitants.^10^ In addition to the imbalance in the proportion of professionals to inhabitants, it was found that there were only ten institutions of higher education with undergraduate dentistry courses regularly authorised by the government, most of which were concentrated in the country’s capital.^10^ Taken together, these findings emphasise the importance of planning, implementing and consolidating policies and strategies that promote the development of human resource training in the dental field capable of adequately meeting the needs of the population.

The present study reveals relevant information about the oral health status of the rural population of Angola, considering the scarcity of comprehensive epidemiological studies focused on this population. Measuring the burden of oral diseases, such as the high prevalence of untreated caries found in this study, can help understand the impact of oral problems on a population’s health and can provide a reliable basis for decision-making for relevant health policies and rational use of limited health resources.^18^

The Global Burden of Disease 2019 study analysed the distribution of the burden of 369 diseases in 21 regions and 204 countries and regions around the world, enabling assessments of the burden and trends of the main oral problems.^7^ Systematic analysis of the Global Burden of Disease 2019 demonstrated that, in the period from 1990 to 2019, there was an increase in the global age-standardized incidence rate of untreated carious lesions in the permanent dentition. Furthermore, the global incidence of untreated caries standardised by age and years lived with disability decreased statistically significantly in absolute numbers, especially in regions with a low sociodemographic index and in populations with greater unmet need for dental services.^12^ These findings corroborate the results of the present study, which found a high prevalence of untreated caries and extremely limited access to dental services.

To address this context of limited access to curative and rehabilitative services, the importance of strategies that shift the focus of efforts by dental professionals and health managers from an approach oriented to curative treatment to an approach oriented towards promotion, prevention and health education, aiming to reduce the incidence of tooth decay. Furthermore, it would be important to promote the integration of oral health prevention programs in partnership with other actions and strategies for the prevention of and education about chronic diseases that have risk factors in common, in addition to the development of effective prevention and control strategies to reduce the prevalence of untreated caries.^12,14^

Therefore, it is highly important to promote actions and strategies that make it possible to identify population groups exposed to the greatest risk of developing tooth decay, aiming to develop prevention, promotion and health education programs focusing on these individuals. Such measures become even more relevant when considering the low access to dental services identified in the investigated population, characterised by the reduced number of restored teeth, and the negative impact of untreated tooth decay in adolescents and adults. The sequelae of untreated tooth decay include impaired quality of life related to oral health, pain, infections, decreased chewing efficiency, loss of dental elements, decreased learning performance and increased risk of mortality.^9,11,13,20^ These consequences are severe, and must be considered in the planning stages of public health policies aimed at improving the population’s oral health.

A worrying situation was found regarding the high proportion of individuals who do not use toothpaste to clean their mouths. Many studies have shown that regular toothbrushing with fluoridated toothpaste is one of the main non-professional interventions for preventing tooth decay, especially among populations in situations of greater vulnerability.^16,21^ Therefore, the findings of this study highlight the need to implement public health strategies and policies that raise awareness among the population about the importance of regularly using fluoride toothpastes and the need to enable a continuous supply of the product, considering the cultural, social and economic context.

In relation to periodontal condition, it is suggested that periodontal disease does not represent a severe public health problem in the population investigated, considering that in all age groups examined, there was a predominance of healthy oral sextants or with clinical conditions of gingival bleeding or dental calculus. Gingivitis, characterised by inflammation of the gums due to the accumulation of bacteria and debris between the gums and teeth, is an initial and milder form of periodontal disease and can be reversed by improving oral hygiene and prevented through adequate maintenance of frequent oral hygiene, carried out by the individual and, when necessary, complemented by professional action to remove dental biofilm.^3,8^

The study findings revealed that, among the individuals who had attended the dentist, the vast majority had had their last dental appointment at a public health/dental service and that a considerable proportion considered the quality of the care received to be poor. As a possible measure to ameliorate this dissatisfaction, educational institutions that train human resources in the dental area could play a prominent role in developing initiatives to improve access to oral health care for populations in vulnerable situations, for instance, through projects integrated with the governmental and local health services, promoting educational, preventive and curative interventions to meet the needs of the population.^15^

The study was conducted in a rural community in the province of Benguela, Angola, and its findings may not exactly reflect the situation of rural populations in other regions of the country, which can be considered a limitation of the study. Nevertheless, given the scarcity of data on the oral health condition of rural populations of Angola, the present study can make an important contribution to the planning of strategies and actions aimed at the main needs of this population.

## CONCLUSION

Adolescents and adults in rural areas of Angola have an extreme lack of access to dental services, mainly limited to extraction interventions. The prevalence of untreated caries is high, while periodontal disease has not proven to be a severe problem in this population.

## ACKNOWLEDGMENTS

This study was financed in part by the Coordenação de Aperfeiçoamento de Pessoal de Nível Superior – Brasil (CAPES) – Finance Code 001.
